# Endoscopy-assisted endotracheal intubation for advanced interventional procedures requiring general anesthesia

**DOI:** 10.1055/a-2422-5887

**Published:** 2024-10-14

**Authors:** Jingjing Yao, Yongbin Han, Lei Kong, Wenwen Hou, Qiuzi Yang, Jindong Fu

**Affiliations:** 1 549615 Department of Gastroenterology, Rizhao Peopleʼs Hospital, Rizhao, China; 2 549615 Department of Anesthesiology, Rizhao Peopleʼs Hospital, Rizhao, China

In complex upper gastrointestinal tract endoscopic submucosal dissection (ESD) procedures, prolonged operations increase the risk of bleeding and patient aspiration. To mitigate these risks and ensure patient safety, tracheal intubation is often required 1 . Here, we report an endoscopy-assisted tracheal intubation technique that enhances safety and efficiency ( Video 1 ).

Endoscopy-assisted tracheal intubation is performed to enhance the safety and efficiency of the procedure.Video 1

The patient is positioned in a left lateral decubitus position, with a bite block in place. After the induction of general anesthesia and mask ventilation, oxygen is administered for 3–5 minutes to denitrogenate the lungs. The anesthesiologist shapes the tracheal tube with an inserted stylet, creating a 70–80° angle at the cuff area ( Fig. 1 ). Under direct endoscopic vision, the glottis is exposed. The preshaped tracheal tube is inserted into the oral cavity from the patientʼs right side. Once the cuff is fully inside the oral cavity, the tube is adjusted to the midline sagittal position; the tip of the tube is now visible in front of the glottic opening on endoscopic view ( Fig. 2 **a** ). With a steady endoscopic view, the tube is rotated to place the tip into the glottis ( Fig. 2 **b** ). The stylet is then removed while simultaneously advancing the tube to the appropriate depth. The cuff is inflated, and the anesthetic machine is connected for mechanical ventilation. Concurrently, the gastroscope is advanced to perform the relevant endoscopic treatment.

**Fig. 1:**
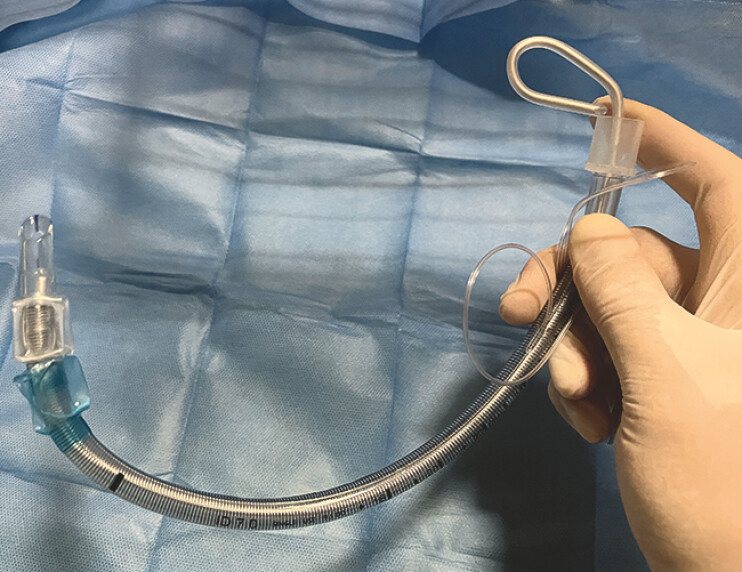
Photograph of the tracheal tube with a stylet inserted to preshape it, with the cuff area bent to an angle of 70–80°.

**Fig. 2:**
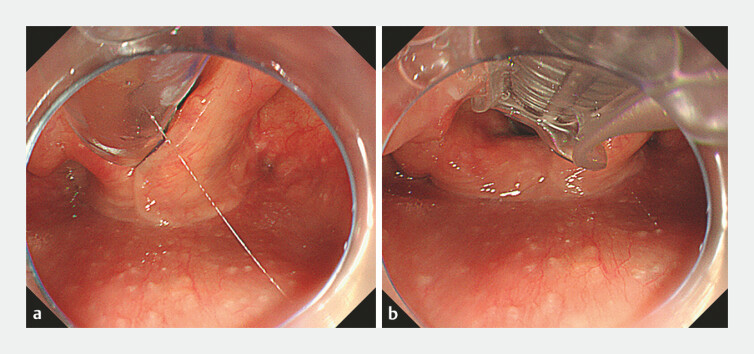
Endoscopic images showing: **a** the tip of the tracheal tube in front of the glottic opening; **b** the tip of the tracheal tube placed into the glottis under endoscopic vision.

Endoscopy-assisted tracheal intubation offers several advantages: First, it eliminates the need to reposition the patient postintubation, thereby reducing the risk of cervical spine injury. Second, it avoids the necessity of placing a bite block after intubation, so preventing potential damage such as tooth loosening. Third, direct endoscopic visualization ensures clearer exposure and expedites the operation process. Endoscopic assistance can present a more convenient and safer approach compared with standard intubation techniques.

Endoscopy_UCTN_Code_TTT_1AU_2AF

## Notes

Endoscopy E-Videos https://eref.thieme.de/e-videos
*E-Videos* is an open access online section of the journal *Endoscopy* , reporting on interesting cases and new techniques in gastroenterological endoscopy. All papers include a high-quality video and are published with a Creative Commons CC-BY license. Endoscopy E-Videos qualify for HINARI discounts and waivers and eligibility is automatically checked during the submission process. We grant 100% waivers to articles whose corresponding authors are based in Group A countries and 50% waivers to those who are based in Group B countries as classified by Research4Life (see: https://www.research4life.org/access/eligibility/ ). This section has its own submission website at https://mc.manuscriptcentral.com/e-videos .
